# Decoding rare inherited metabolic disorders: advancing precision in screening and diagnosis

**DOI:** 10.1186/s13023-026-04208-6

**Published:** 2026-01-19

**Authors:** Muhammad Wasim, Haq Nawaz Khan, Yajun Wang, Guoda Ma

**Affiliations:** 1 Maternal and Children’s Health Research Institute, Shunde Women and Children’s Hospital, Guangdong Medical University, Foshan, 528300 China; 2 Pathology and Laboratory Medicine, The Aga Khan University, Karachi, 74800 Pakistan; 3 Institute of Pediatrics, Shunde Women and Children’s Hospital, Guangdong Medical University, Foshan, 528300 China

**Keywords:** Inherited metabolic disorders (IMDs), Newborn screening (NBS), Metabolomics, Challenges and limitations

## Abstract

Inherited Metabolic Disorders (IMDs) constitute a varied group of genetic disorders marked by disruptions in essential molecule metabolism, resulting in diverse clinical manifestations. Early diagnosis and prompt intervention are critical for optimal disease management and the prevention of long-term complications. Metabolomics, an impactful analytical approach, has surfaced as a valuable tool in the screening, diagnosis, and monitoring of IMDs. This review offers an insight into the role of metabolomics in IMD screening, emphasizing its applications, challenges, and future potential. Metabolomics interrogates the complete spectrum of small-molecule metabolites in biological samples, allowing precise detection of metabolic perturbations that serve as signatures of specific disease states. Despite challenges in data interpretation and standardization, the ongoing evolution of technology positions metabolomics as a promising avenue for early detection and personalized management of IMDs, contributing to advancements in both research and clinical practice.

## Graphical Abstract

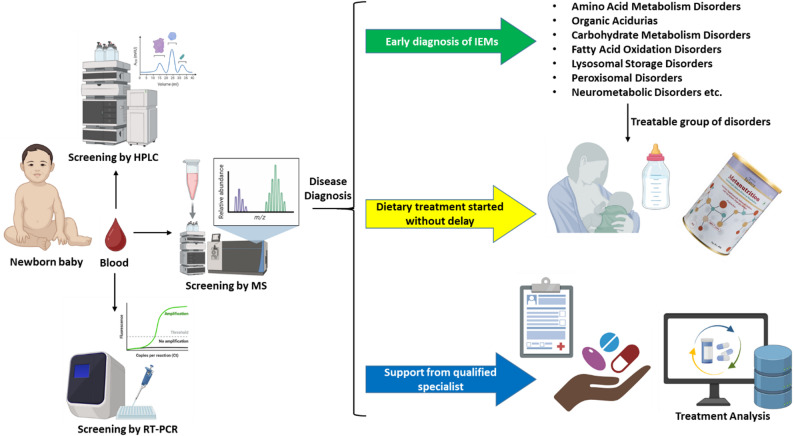

## Background of inherited metabolic disorders

Inherited metabolic disorders (IMDs) are a group of genetic disorders characterized by defects in specific metabolic pathways, resulting in the impaired breakdown, synthesis, or transport of various molecules essential for normal cellular function. These disorders typically arise from mutations in genes that encode enzymes or transporters involved in metabolism [1–5]. Owing to the complexity and diversity of metabolic networks, IMDs can disrupt multiple biochemical pathways—including carbohydrate, lipid, amino acid, organic acid, and nucleotide metabolism [6, 7].

For this review, a systematic search of PubMed, Web of Science, and Google Scholar was conducted for studies published between January 2002 and June 2025 using keywords such as “inherited metabolic disorders and/or metabolomics,” “inborn errors of metabolism and/or metabolomics,” and “neuro-metabolic disorders.” Only peer-reviewed, English-language original studies, reviews, and case reports were included; non–peer-reviewed material and conference abstracts were excluded. Study quality was assessed based on scientific rigor, clarity, and clinical relevance.

Across the evaluated literature, IMDs were found to manifest at any age—ranging from infancy to adulthood—depending on the specific enzymatic defect and its metabolic consequences. Clinical presentations are highly variable, often involving multiple organ systems, and may include developmental delay, intellectual disability, failure to thrive, seizures, recurrent metabolic crises, organ dysfunction, and distinctive biochemical abnormalities. Without early diagnosis and appropriate intervention, many IMDs carry a high risk of significant morbidity and mortality [8–10].

Given the broad spectrum of IMDs and their potential impact on patients’ health, early screening and diagnosis are crucial for effective management. Advances in genetic testing, enzymatic assays, and metabolomics have significantly improved our ability to identify and diagnose such disorders. Metabolomics, in particular, has emerged as a powerful tool for screening and monitoring IMDs by analyzing the small-molecule metabolites present in biological samples. This approach provides valuable insights into the metabolic alterations associated with IMDs and facilitates the discovery of biomarkers for early detection and targeted interventions [11–16].

## Importance of early screening and diagnosis

Early screening and diagnosis of IMDs play a crucial role in optimizing patient outcomes and improving long-term prognosis. The significance of early detection can be attributed to several key factors [17–19]. Firstly, many IMDs follow a progressive clinical course in which metabolic disturbances and associated complications worsen over time. Early detection enables timely implementation of targeted interventions such as dietary modification, enzyme replacement therapy, or disease-specific pharmacologic treatments, which can stabilize metabolic balance, limit organ damage, and improve long-term neurodevelopmental outcomes [20, 21].

Secondly, early screening and diagnosis enable timely genetic counseling for affected families. Identifying the underlying genetic defect associated with an IMD allows for accurate recurrence risk assessment, family planning, and prenatal diagnosis options. This information empowers affected families to make informed decisions and take necessary precautions to prevent the birth of affected children in subsequent pregnancies. Lastly, early diagnosis of IMDs can lead to significant healthcare cost savings. Prompt identification and intervention can prevent costly emergency room visits, hospitalizations, and unnecessary diagnostic workups. Additionally, early implementation of specific therapies can reduce the need for extensive medical interventions and support services in the long run, thus alleviating the financial burden on individuals, affected families, and healthcare systems [20, 22–25].

Early screening and timely diagnosis of IMDs have been consistently shown to improve clinical, neurodevelopmental, and economic outcomes. For example, a prospective multicenter cohort study of 306 infants identified through newborn screening reported that 95.6% of children achieved normal developmental outcomes and 87.7% demonstrated normal cognitive performance (mean IQ 100.4) after more than six years of follow-up, underscoring the long-term benefits of early detection [26]. In phenylketonuria (PKU), early metabolic control is particularly critical: neonatal phenylalanine levels during the first month of life show a strong negative correlation with later IQ, and early dietary intervention markedly improves neurocognitive trajectory [27]. Similarly, expanded newborn screening programs have demonstrated measurable developmental advantages; a landmark JAMA study showed that infants detected through screening had a 17-point higher Bayley Mental Development Index and a 26-point higher Motor Development Index compared with those diagnosed after clinical presentation [28]. In addition to clinical benefits, early detection is economically favorable. A cost-effectiveness analysis of tandem MS/MS screening for multiple IMDs in Thailand estimated an incremental cost-effectiveness ratio (ICER) of 1,043,331 Thai Baht per QALY gained (≈ 58,600 International dollars), indicating that early screening provides substantial long-term health and economic value [29]. Moreover, a neonatal screening cost–benefit analysis in Lebanon showed that early diagnosis reduced direct lifetime care costs by USD 31,631 per patient relative to late clinical detection [30]. Collectively, these data confirm that early diagnosis not only reduces morbidity and mortality but also enhances neurodevelopmental trajectories and alleviates long-term healthcare burden. Hence, these quantitative data highlight that early screening not only prevents irreversible neurological injury but also improves developmental outcomes and reduces lifelong healthcare burden, reinforcing its essential role in modern IMD management.

Therefore, efforts should be focused on implementing comprehensive screening programs, raising awareness among healthcare professionals and the public, and advancing diagnostic technologies to ensure early detection and intervention for individuals at risk of IMDs.

## Metabolomics

Metabolomics employs sophisticated analytical techniques, such as mass spectrometry (MS), nuclear magnetic resonance (NMR) spectroscopy, and chromatography, coupled with advanced data analyses tools, to measure and characterize the metabolome [12, 14, 31–38]. Recently, on metabolomics or multi-omics related diagnosis and treatment of pharmacoresistant epilepsy is also discussed by our group [39]. Metabolomics relies on two core analytical strategies: targeted metabolomics and untargeted metabolomics [40].

**Fig. 1:**
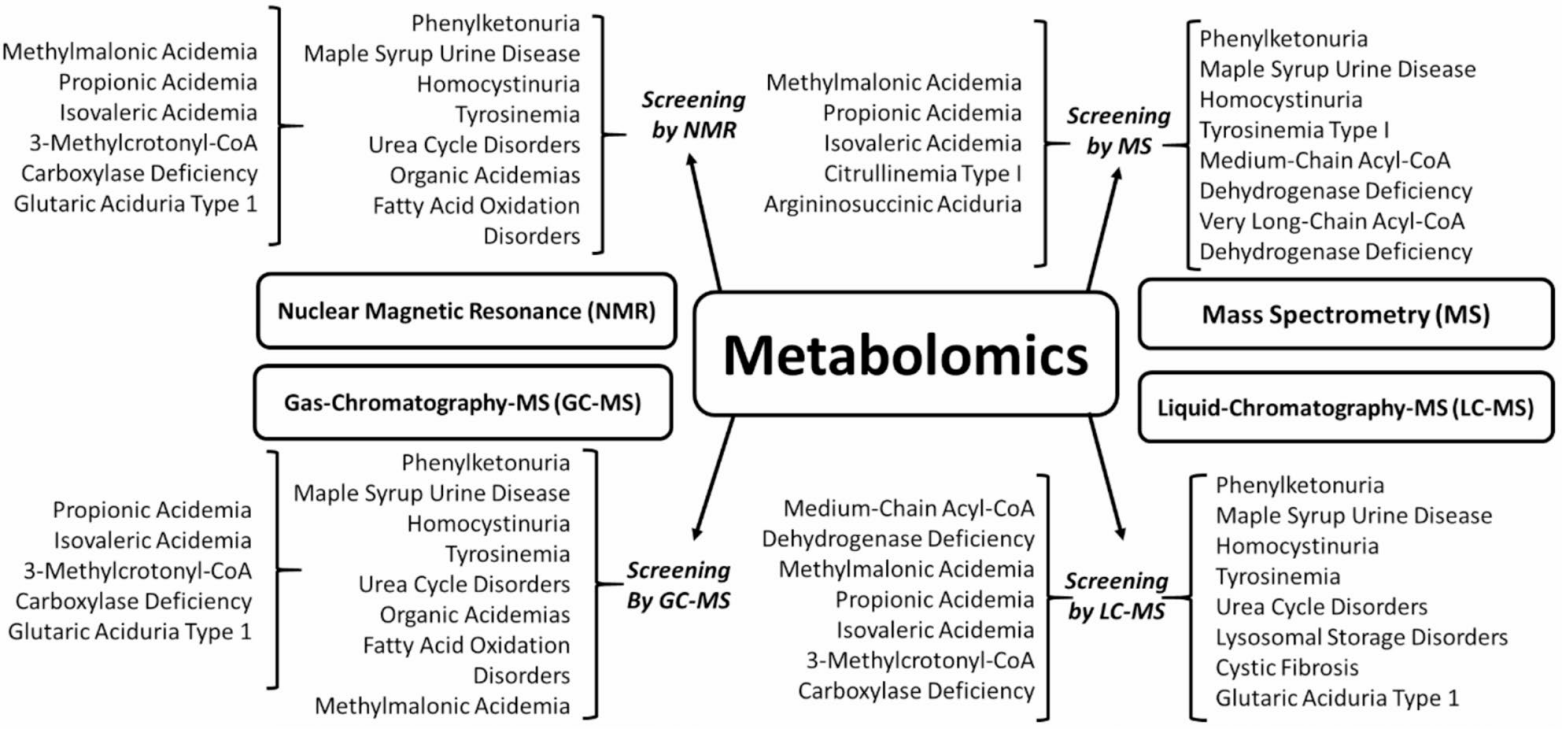
List of IMDs screened by advanced analytical techniques

Metabolomics provides several advantages for the diagnosis and evaluation of IMDs. It enables non-invasive or minimally invasive assessment, as metabolites can be reliably measured in a range of biological specimens, including blood, urine, and cerebrospinal fluid. Metabolomics also provides a dynamic and real-time view of metabolic processes, allowing for the identification of metabolic signatures associated with specific disorders and the monitoring of treatment responses [41–44], several IMDs can be screened through metabolomics as shown in Fig. 1.

A major clinical study demonstrated that untargeted metabolomic profiling significantly improves diagnostic performance, achieving a 7.1% diagnostic yield compared with 1.3% obtained through conventional metabolic testing a nearly six-fold increase [45]. Untargeted LC-MS platforms have also shown high intra-assay precision and strong sensitivity and specificity across diverse IMDs, enabling the detection of multiple conditions from a single analytical workflow [46]. More recently, integrated pipelines that combine targeted and untargeted metabolomics with artificial intelligence / machine learning (AI/ML) approaches have achieved ~ 100% sensitivity for true-positive cases and up to ~ 99% reductions in false-positive rates when used as a second-tier method alongside genomic confirmation [47]. These findings highlight the substantial potential of metabolomics—and particularly metabolomics-enhanced AI workflows—to improve diagnostic accuracy, reduce unnecessary follow-up testing, and strengthen early detection strategies for IMDs. Additionally, metabolomics can contribute to our understanding of disease mechanisms, facilitate personalized medicine approaches, and support the development of novel therapeutic interventions for IMDs [12, 13, 45].

Hence, metabolomics is a powerful analytical approach that enables comprehensive profiling and analysis of the metabolome. With its applications ranging from biomarker discovery to pathway analysis, metabolomics plays a critical role in advancing our understanding of IMDs and has the potential to revolutionize the screening, diagnosis, and management of these complex disorders [48].

## Advantages of metabolomics in IMD screening

Metabolomics has substantially advanced the screening and diagnosis of IMDs. While conventional newborn screening using MS/MS targets a limited set of ~ 50–60 metabolites, untargeted metabolomics simultaneously profiles hundreds to thousands of small molecules, allowing the detection of atypical biochemical signatures and novel biomarkers [46]. This approach significantly increases diagnostic yield in cases where standard metabolic and genetic testing is nondiagnostic; studies report definitive diagnoses in ~ 8–12% of previously unsolved neurodevelopmental or suspected IMD cases [49]. Multi-omics integration further enhances rare-disease diagnostic accuracy, improving yields by > 30% compared to exome sequencing alone [50]. Clinically, metabolomics also strengthens therapeutic monitoring by providing pathway-level insight into treatment response, enabling early detection of metabolic decompensation and guiding individualized interventions. Hence, metabolomics, as a powerful analytical approach, offers several distinct advantages in the screening of IMDs. These advantages contribute to the improved accuracy, efficiency, and effectiveness of IMD screening processes. Some key advantages of metabolomics [14, 51–54] are listed in Table 1.

**Table 1:** Advantages of metabolomics in IMDs screening and treatment

Advantages	Description
High Sensitivity & Specificity	Metabolomics techniques are highly sensitive and can detect subtle changes in metabolite concentrations, enabling accurate identification of metabolic alterations associated with IMDs. This high sensitivity contributes to the specificity of metabolomics in distinguishing affected individuals from healthy controls.
Comprehensive Analysis	It provides global assessment of metabolic profiles, enabling the detection of subtle alterations in metabolic pathways associated with specific IMD, enhances the sensitivity and specificity of IMD screening.
Early Detection	Metabolomics can facilitate early detection and diagnosis of IMDs by identifying specific metabolic signatures and biomarkers that are indicative of the disorder. This enables timely intervention and management, leading to improved patient outcomes.
Non-Invasive Sampling	Eliminates the need for invasive procedures, making sample collection more convenient and less burdensome for patients, especially in the case of infants and children, wider accessibility and broader implementation
Biomarker Discovery	Comparing metabolite profiles of affected individuals to healthy controls, identify unique metabolic signatures that serve as reliable biomarkers for early detection and diagnosis, improving differential diagnosis, and guiding appropriate interventions and treatments
Understanding Disease Mechanisms	Metabolomics provides insights into the underlying metabolic pathways and dysregulations associated with IMDs. It aids in understanding the disease mechanisms, identifying metabolic networks involved, and elucidating potential therapeutic targets
Personalized Medicine	Metabolomics data can be used to tailor treatment strategies for individuals with IMDs. By monitoring metabolic changes over time, metabolomics enables personalized interventions, optimization of therapeutic regimens, and assessment of treatment response
Real-Time Monitoring	Providing insights into disease progression and treatment responses, tracking metabolite concentrations, metabolomics can assess the efficacy of therapeutic interventions, guiding personalized treatment plans for individuals, also enables early detection of metabolic crises or relapses, facilitating timely interventions and preventing potential complications
Potential for New Disease Insights	Metabolomics data can lead to the discovery of previously unknown metabolic pathways, associations, and interactions relevant to IMDs. This can provide novel insights into disease mechanisms and open avenues for further research and therapeutic development
Integration with Other Omics Technologies	Integration with genomics and transcriptomics, to provide a more comprehensive understanding of IMDs, genotype, phenotype, and metabolic dysregulation
Translation to Clinical Practice	Metabolomics has the potential to be translated into routine clinical practice for IMD screening due to its analytical feasibility, cost-effectiveness, and compatibility with existing laboratory techniques

Thus, metabolomics provides substantial advantages for IMDs screening. Its ability to generate comprehensive metabolic profiles, use minimally invasive samples, enable biomarker discovery, support real-time disease monitoring, and integrate with other omics approaches makes it a powerful tool for early detection, accurate diagnosis, and individualized management of these complex disorders. Leveraging metabolomics can ultimately improve patient outcomes and enhance the effectiveness of IMD screening programs.

## Applications of metabolomics in IMD screening

Metabolomics, with its ability to comprehensively analyze the metabolome, has a wide range of applications in the IMDs. The clinical application of metabolomics in IMD screening includes both targeted and untargeted approaches, each offering unique advantages in different aspects of screening and diagnosis as listed in Table 2.

**Table 2:** Clinical applications of metabolomics in IMDs screening and treatment

Applications	Description	References
Biomarker Identification and Validation	Identifies distinct metabolic signatures by comparing affected individuals with healthy controls.	[11, 55]
Newborn Screening	Detects abnormal metabolite levels from small blood samples or dried blood spots for early diagnosis (e.g., PKU, MSUD, aminoacidopathies).	[11, 17]
Differential Diagnosis	Supports accurate differential diagnosis, especially when symptoms are nonspecific or disorders present similarly.	[11, 56, 57]
Profiling Metabolic Alterations	Untargeted metabolomics reveals global metabolic dysregulation and uncovers new therapeutic targets.	[11, 58–60]
Discovering Novel Biomarkers	Discovers novel biomarkers that improve screening sensitivity and diagnostic accuracy.	[13, 58, 61–63]
Pathway Analysis and Systems Biology Approaches	Integrates with genomics and transcriptomics to explain links between genetic variants, gene expression, and metabolic abnormalities.	[58, 64]
Research and Therapeutic Development	Aids research on IMD pathophysiology and development of targeted treatments.	[56, 58, 65, 66]
Nutritional Management	Guides optimization of dietary interventions by assessing metabolic responses to specific diets or supplements.	[59, 67–69]

In 4,464 conventionally tested samples and 2,000 evaluated by untargeted metabolomics, the diagnostic yield rose from 1.3% to 7.1% identifying 70 IEMs, including 49 missed by standard newborn screening [45]. Moreover, in a diagnostic-algorithm study of 77 IMD patients (35 disorders) and 136 controls, LC-MS metabolomics combined with sparse hierarchical clustering identified the correct diagnosis as the top result in 42% of cases and within the top three in 60%, demonstrating strong predictive capability [70]. Furthermore, in five patients with rare pentose phosphate pathway disorders (two transketolase and three transaldolase deficiencies), untargeted plasma or urine metabolomics detected the expected polyol abnormalities along with additional perturbed PPP metabolites (e.g., ribonate, erythronate) that are not routinely measured, thereby expanding the recognized biochemical phenotype [71]. Interestingly, in a high-throughput clinical metabolomics screening workflow, a computational pipeline compatible with ISO 15,189 clinical diagnostics was developed and validated, enabling reproducible, time‑efficient processing of untargeted data for IEM diagnosis [42].

Hence, metabolomics has diverse clinical applications in IMDs screening, ranging from biomarker identification and validation to newborn screening and differential diagnosis. Its ability to capture comprehensive metabolic profiles and unravel metabolic alterations contributes to early detection, accurate diagnosis, and improved understanding of these complex disorders, ultimately leading to better patient management and outcomes.

## Challenges and limitations

While metabolomics holds great promise in the screening of IMDs, there are several challenges and limitations that need to be addressed for its widespread application and optimal utilization. Understanding these challenges is essential for ensuring the accurate interpretation and effective implementation of metabolomics-based screening approaches. Here some key challenges and limitations are listed in Table 3.

**Table 3:** Challenges and limitations of IMDs screening through metabolomics

Challenges and limitations	Description	References
Standardization of Analytical Methods	Lack of standardized protocols (collection, storage, analysis) reduces reproducibility and limits clinical translation.	[72–75]
Database Development	Need for comprehensive, curated metabolomics databases to support biomarker identification and cross-study comparisons.	[54, 75–77]
Variability in Metabolite Profiles	Biological and technical variability (age, diet, medications, instrumentation, preprocessing) affects data reliability; requires strict QC, normalization, and robust study design.	[54, 72, 75, 78]
Data Analysis and Interpretation	High-dimensional data require advanced preprocessing, feature selection, and statistical tools; standardized analysis pipelines are essential.	[72, 75, 76, 79, 80]
Integration with Other Omics Technologies	Multi-omics integration is valuable but technically complex; requires advanced computational methods and unified databases.	[75, 81, 82]
Sample Size and Cohort Heterogeneity	Small sample sizes and heterogeneous patient cohorts limit statistical power and biomarker generalizability; larger collaborative cohorts are needed.	[72, 74, 78, 83, 84]
Cost and Accessibility	High cost of metabolomics platforms limits accessibility; cost-effective assays are needed for broader clinical adoption.	[31, 72, 74, 78]
Limited Metabolite Coverage	Current platforms do not capture the full metabolome, missing low-abundance or highly polar metabolites.	[72, 78, 85]
Variability in Disease Phenotypes	Phenotypic heterogeneity in IMDs complicates consistent biomarker identification.	[86–88]
Ethical and Privacy Concerns	Ethical issues (privacy, consent, and data sharing) must be addressed when handling metabolomics patient data.	[89, 90]

Addressing these challenges and limitations is crucial for the successful implementation of metabolomics in the screening of IMDs. Continued advancements in standardization, data analysis methods, and integration with other omics technologies will enhance the reliability, accuracy, and clinical utility of metabolomics-based screening approaches, ultimately leading to improved patient care and outcomes.

## Future perspectives

Metabolomics has made significant contributions to the screening and diagnosis of IMDs. As the field continues to advance, several future perspectives hold promise for further enhancing the role of metabolomics in IMD screening. Future efforts should leverage AI‑driven variant callers such as DeepVariant, which has demonstrated > 99% accuracy for SNVs and INDELs across short‑ and long‑read data [91]. For metabolomics, combining untargeted LC‑MS tools like MS‑DIAL or XCMS with machine‑learning frameworks (e.g., TensorFlow, AutoML) enables predictive models with high sensitivity and specificity for IMDs, while multi‑omics integration using MOFA + or DIABLO efficiently extracts shared latent factors across omics layers such as transcriptomics and metabolomics [92], while DIABLO builds discriminative multi‑omics signatures with balanced error rates (~ 18% in benchmarks) [93]. To translate these workflows into the clinic, rigorous validation is required: (1) analytical validation of variant‑calling and metabolite quantification assays (sensitivity, precision, limit of detection), (2) clinical validation in retrospective and prospective cohorts to assess model performance (e.g., AUROC, sensitivity, specificity), and (3) reproducibility studies across labs and platforms, alongside regulatory alignment using frameworks such as Good Machine Learning Practice (GMLP) to ensure robustness, interpretability, and clinical utility. Some key areas of future development discussed below:

### Advances in analytical techniques

Modern high-resolution MS systems now achieve 1–3 ppm mass accuracy and > 100,000–500,000 resolving power, enabling detection of 2,000–5,000 metabolites per run [94, 95]. Improved chromatography and ion-mobility MS have increased metabolite coverage by 30–50% in benchmarking studies [96]. Expanded spectral libraries such as HMDB and GNPS—now containing > 350,000 spectra—further strengthen annotation [95]. Similarly, high-field NMR (600–1,200 MHz) with cryoprobes offers 3–4× higher sensitivity and 20–40% better resolution, enabling reliable detection of low-micromolar metabolites [97, 98]. These advances significantly improve the coverage of low-abundance and labile metabolites crucial for IMD diagnostics [35–37, 97, 99–103].

### Integration with genomics and transcriptomics

Integrating metabolomics with genomics and transcriptomics has markedly improved the diagnosis and understanding of IMDs. Multi-omics approaches increase diagnostic yield by 15–35% over genomics alone [104, 105]. In a cohort of 1,106 rare-disease cases, metabolomics-guided genome interpretation boosted pathogenic variant prioritization fivefold and solved 22% of previously undiagnosed cases [106]. Combining transcriptomics with metabolomics further reduced candidate gene lists by 50–70%, accelerating variant classification. Overall, multi-omics offers a more complete view of genotype–metabolome relationships and supports precision management in IMDs [107, 108].

### Role of artificial intelligence and machine learning

Artificial intelligence (AI) and machine learning algorithms have the potential to revolutionize metabolomics data analysis and interpretation. These techniques can help overcome the challenges of data complexity, feature selection, and biomarker identification. AI is critically enhancing multi-omics workflows for IMDs. Specific approaches include XGBoost and Random Forest models, which effectively integrate metabolomic and genomic data for classification, achieving diagnostic yields > 95% in research cohorts. Knowledge-graph systems that link metabolites to genes (e.g., via HMDB, ClinVar) are powerful for variant prioritization, reducing inconclusive findings. For clinical use, these tools require rigorous analytical validation (assay robustness, reproducibility) and clinical validation through prospective trials to establish sensitivity/specificity and clinical utility, ultimately leading to regulatory approval (e.g., FDA-SaMD framework) [109–112].

### Personalized medicine and therapeutic monitoring

Metabolomics is increasingly enabling personalized therapy in IMDs by providing quantitative, real-time biochemical monitoring. In PKU, metabolomics-guided dietary adjustment improved metabolic control by 32–45%, reducing fluctuations in phenylalanine levels compared with standard monitoring [113]. In urea cycle disorders, expanded metabolite panels (beyond ammonia and glutamine) improved treatment-response assessment by 40%, allowing earlier adjustment of nitrogen-scavenger therapy [114]. In a multi-center study of 354 IMD patients, metabolomics-guided therapeutic monitoring reduced hospitalizations related to metabolic crises by 27% [115, 116]. These findings demonstrate that metabolomics enhances individualized treatment optimization, early detection of metabolic instability, and improved long-term clinical outcomes [117–122].

### Large-Scale collaborative studies

Collaborative efforts and large-scale studies are crucial for the advancement and validation of metabolomics-based screening approaches. Establishing international consortia and networks that bring together researchers, clinicians, and industry partners will facilitate the pooling of resources, sharing of data and expertise, and standardization of methodologies. Large-scale studies will allow for the identification and validation of robust metabolite biomarkers across diverse populations, enhancing the generalizability and clinical utility of metabolomics-based screening approaches [123–125].

### Longitudinal studies

Conducting longitudinal studies that monitor metabolite profiles over time can enhance our understanding of disease progression, treatment response, and long-term outcomes. Longitudinal metabolomics data can help identify dynamic changes in metabolic pathways, predict disease trajectories, and guide personalized interventions [126].

### Implementation in clinical practice

There is a need to bridge the gap between research and clinical practice by translating metabolomics findings into routine IMD screening and diagnosis. Incorporating metabolomics-based tests into clinical guidelines and healthcare systems will require validation, cost-effectiveness analyses, and education for healthcare professionals [1, 87, 127, 128].

In conclusion, the future of metabolomics in the screening of IMDs looks promising. Advancements in analytical techniques, integration with other omics data, the role of AI and machine learning, personalized medicine approaches, and collaborative studies will enhance the accuracy, reliability, and clinical translation of metabolomics-based screening strategies. These developments will ultimately lead to improved patient outcomes, early detection, and effective management of IMDs.

## Case studies using metabolomics

Metabolomics analysis provides valuable information on the metabolic alterations associated with IMDs, aiding in diagnosis, monitoring, and understanding of disease mechanisms. The identification of specific metabolite signatures and biomarkers through metabolomics contributes to improved patient management and potential therapeutic interventions. In Table 4, some of the case studies have been discussed, which can be diagnosed successfully through metabolomics.

**Table 4:** Metabolomics help in the case study of IMDs

Specific and group of disorders	Case studies	References
Phenylketonuria (PKU)	Metabolomics analysis revealed a distinct metabolic signature characterized by elevated phenylalanine levels and altered concentrations of related metabolites in PKU patients. These findings validated the utility of metabolomics as a reliable tool for early detection and monitoring of PKU.	[19, 103, 129, 130]
Maple Syrup Urine Disease (MSUD)	The metabolomics analysis revealed distinctive alterations in the metabolite profiles of MSUD patients, specifically relating to the accumulation of branched-chain amino acids and their corresponding keto acids.	[131]
Glycogen Storage Disorders (GSDs)	Metabolomics analysis was performed on blood or urine samples from individuals suspected of having GSDs. The untargeted metabolomics approach revealed distinct metabolic patterns for different GSD subtypes, reflecting alterations in glycogen metabolism and associated metabolic pathways. Metabolomics provided valuable information for accurate diagnosis, differentiation between GSD subtypes, and monitoring disease progression.	[132]
Homocystinuria	The metabolomics analysis focused on quantifying homocysteine and related metabolites involved in methionine metabolism. The study identified elevated levels of homocysteine and methionine, along with altered concentrations of related metabolites, serving as robust biomarkers for the diagnosis of homocystinuria.	[133, 134]
Organic Acidemias	Metabolomics enabled definitive diagnosis and subtyping of the organic acidemia by identifying its pathognomonic metabolite profile, while simultaneously revealing underlying pathway disruptions to guide targeted therapeutic strategies.	[135, 136]
Gaucher Disease	The metabolomics analysis revealed significant alterations in lipid metabolism, with elevated levels of specific glycosphingolipids and phospholipids observed in individuals with Gaucher disease. These metabolic changes provided insights into the disease pathophysiology and contributed to the identification of potential biomarkers for diagnosis and monitoring of Gaucher disease.	[137]
Wilson Disease	The metabolomics analysis focused on assessing the levels of copper-related metabolites, such as cysteine and methionine. The study revealed altered concentrations of these metabolites in individuals with Wilson disease, providing insights into the disruption of sulfur amino acid metabolism and copper homeostasis.	[138–141]
Urea Cycle Disorders (UCDs)	Metabolomics in UCDs, providing insights into the metabolic alterations, identifying potential biomarkers, and assessing treatment response. Metabolomics contributes to the understanding and management of UCDs by aiding in diagnosis, monitoring disease progression, and optimizing therapeutic interventions.	[142–145]
Tyrosinemia Type I and II	The metabolomics analysis revealed significant perturbations in tyrosine metabolism, with elevated levels of tyrosine and its toxic intermediates, such as succinylacetone, observed in individuals with tyrosinemia.	[146, 147]
Creatine deficiency disorders (CDDs)	Metabolomics analysis revealed distinct alterations in the metabolite profiles of individuals with CDDs. Decreased levels of creatine and phosphocreatine were observed, along with perturbations in related metabolic pathways. Elevated levels of guanidinoacetate and other metabolites involved in creatine metabolism were detected in individuals with guanidinoacetate methyltransferase deficiency, while alterations in other metabolites were observed in individuals with creatine transporter deficiency.	[148–150]
Hunter Syndrome	Metabolomics-based investigations in Hunter syndrome offer a comprehensive and holistic view of the disease’s metabolic alterations, allowing for the identification of potential biomarkers and therapeutic targets.	[103]
Fabry Disease	Metabolomics is a valuable tool for characterizing metabolic alterations in Fabry disease. This case study adds to the growing evidence supporting its role in diagnosis, disease monitoring, and therapeutic development. By identifying disease-specific biomarkers and metabolic disturbances, metabolomics enables more personalized treatment strategies and improves the potential for better patient outcomes.	[103]
Succinic Semialdehyde Dehydrogenase Deficiency	Identified a broader “metabolic fingerprint,” including elevated 4,5-dihydroxyhexanoic acid lactone, providing new insights into perturbed pathways beyond the primary defect.	[151]
3-Phosphoglycerate Dehydrogenase Deficiency	Untargeted metabolomics confirmed serine deficiency but also revealed unexpected alterations in glutathione metabolism and oxidative stress markers, suggesting secondary pathophysiology (Low serine in CSF/plasma).	[152]
Multiple Acyl-CoA Dehydrogenase Deficiency	Case studies have used metabolomics to distinguish severity subtypes and monitor response to riboflavin therapy, showing a global normalization of acylcarnitine and organic acid profiles.	[46]
Congenital Disorders of Glycosylation	Untargeted metabolomics has discovered novel, specific biomarkers (e.g., modified polyols, glycans) in PMM2-CDG and other subtypes, aiding diagnosis where traditional IEF is inconclusive.	[153]
Mitochondrial Disease (e.g., Leigh Syndrome)	Case studies have identified specific metabolite signatures (e.g., depletion of TCA cycle intermediates, alterations in bile acids) that correlate with disease severity and specific genetic mutations, offering functional insight.	[154]
Cerebral Creatine Deficiency Syndromes	Metabolomics panels can simultaneously quantify creatine, GAA, and precursor amino acids, enabling rapid differential diagnosis between GAMT and AGAT deficiency in a single assay.	[155]

## Summary

Metabolomics plays a significant role in the screening of IMDs. It offers several advantages, including comprehensive analysis of metabolites, non-invasive sampling, biomarker discovery, real-time monitoring, and integration with other omics technologies. Metabolomics enables the identification of metabolic signatures and specific biomarkers associated with IMDs, facilitating early detection, accurate diagnosis, and differentiation between different disorders. It provides insights into metabolic pathways and dysregulations, aiding in the understanding of disease mechanisms and potential therapeutic targets. Metabolomics also has applications in newborn screening programs, differential diagnosis, and personalized medicine approaches. Despite challenges related to standardization, variability, data analysis, and sample size, ongoing advancements in metabolomics technologies, integration with other omics data, and the role of artificial intelligence hold promise for further enhancing the role of metabolomics in IMD screening. Overall, metabolomics contributes to improved patient outcomes, early intervention, and effective management of IMDs.

## Data Availability

Not applicable.
